# Serum 25-Hydroxyvitamin D were associated with higher risk of both albuminuria and impaired GFR incidence: a cohort study based on CLHLS study

**DOI:** 10.1186/s12882-019-1202-8

**Published:** 2019-01-15

**Authors:** Miao Liu, Jianhua Wang, Yao He

**Affiliations:** Institute of Geriatrics, Beijing Key Laboratory of Aging and Geriatrics, National clinical research center for geriatrics diseases, State Key Laboratory of Kidney Diseases, Chinese PLA General Hospital, 28 Fuxing Road, Beijing, 100853 China

**Keywords:** Chronic kidney disease, Impaired glomerular filtration rate, Albuminuria 25-Hydroxyvitamin D, Cohort

## Abstract

**Background:**

This study aimed to examine the relationship between 25-hyfromxyvitamin D (25OHD) and chronic kidney disease (CKD) incidence.

**Methods:**

All the elderly who had participated both in the 2011–2012 survey and 2014 survey in the Chinese Longitudinal Healthy Longevity Survey (CLHLS), and have biomarker data were included in the analysis. We studied those without CKD with complete data at 2011–2012 waves. Serum 25-Hydroxyvitamin D was assessed at baseline. Cox proportional risk model was used to evaluate associations between serum 25-Hydroxyvitamin D and CKD (including both albuminuria and impaired eGFR) incidence after adjusted for potential confounding..

**Results:**

During the follow-up years, 255 incident cases of CKD were diagnosed. Those who developed CKD had relatively lower serum 25(OH)D (mean 37.63 vs.51.36 nmol/L, *p* < 0.001) compared with those who remained free of CKD. Each 1 nmol/L increase in 25(OH)D was associated with 3.4% reduced risk of CKD (HR = 0.966, 95%CI: 0.959–0.973) after adjusted for related covariates. The HRs of each 1 nmol/L increase in 25(OH)D for albuminuria and impaired eGFR were 0.952(95%CI: 0.941–0.963) and 0.975(95%CI: 0.966–0.983) respectively. When use the classifications (sufficiency, insufficiency, deficiency) or quintiles of baseline 25(OH)D levels in the Cox model, the corresponding HRs showed an increasing trend along with the decrease of baseline 25(OH)D levels (p for trend < 0.001).

**Conclusions:**

Higher 25(OH)D levels were inversely and independently associated with CKD incidence among Chinese elderly. The trend for the observed linear relationship b was most pronounced among the lowest quintile.

**Electronic supplementary material:**

The online version of this article (10.1186/s12882-019-1202-8) contains supplementary material, which is available to authorized users.

## Background

Vitamin D is a pro-steroid hormone, and first hydroxylated to 25 (OH) D in the liver, and further hydroxylated to 1,25(OH)_2_D in the renal proximal convoluted tubules. Then 1,25(OH)_2_D were combined with receptors in the target tissues to play its biological roles [1]. The widespread recognition of vitamin D was its regulation of calcium and phosphorus metabolism. In recent years, it has been found that vitamin D has a wide range of metabolic and cell regulatory functions [2]. It has been a recent research hotspot about the role of vitamin D in tumor, endocrine, immunity, cardiovascular and kidney diseases [3–5]. It plays an important role in inhibiting cell proliferation, inducing cell apoptosis and differentiation, regulating immune function, protecting organ function and gene protection by combining with vitamin D receptor [6].

Chronic kidney disease (CKD) is one of the most important diseases which affect about 10–15% of adults. Epidemiological data showed that the prevalence of CKD increase for the past 20 years [7, 8]. Besides, compared with cardiovascular diseases and diabetes, people have not paid sufficient attention to CKD. Most of the patients went to the hospital when they had appeared clinical symptoms or were already in the late stage, resulting in poor prognosis and huge disease burden [9]. Therefore, there is a series of researches focused on risk factors about CKD incidence. Recently, a series of studies showed that vitamin D and serum 25(OH)D deficiency had emerged as one important independent risk factor for CKD [4, 10, 11]. Animal and cell cultural experimental data showed that vitamin D inhibits renin transcription, reduces circulating angiotensin II level, prevents phagocyte loss and glomerulosclerosis, and reduces urinary albumin excretion. And in human studies, lower vitamin D levels were independently associated with higher risk of albuminuria and CKD. However, most of the studies about vitamin D and CKD were cross-sectional studies, and there were lack of evidence based on prospective studies. In addition, the prevalence of vitamin D deficiency varies by gender, season of the year, sunlight exposure and a number of other environmental factors and comorbidities [12]. Besides, most studies were of albuminuria, less were about the impaired estimated glomerular filtration (eGFR), which was also very important for CKD [4, 8, 10, 13]. Accordingly, this study, which was based on data from two waves of the Chinese Longitudinal Healthy Longevity Survey (CLHLS) occurring from 2011 to 2012 to 2014, aimed to examined weather lower 25(OH)D level was associated with both albuminuria and impaired eGFR based on a cohort design using Cox proportional risk model.

## Methods

### Study population

The data of this study was from CLHLS, a high-quality longitudinal survey based on Chinese elderly [14]. In CLHLS study, a multi-stage target random sampling method with unequal proportion was adopted in order to ensure enough samples for each age group. The sampling steps were as follows: (1) about 50% of the counties/districts were randomly selected from 22 provinces; (2) in these areas, all surviving centenarians who volunteered to participate in the survey were visited by households; (3) those aged 65–79 yrs., 80–89 yrs., and 90–99 yrs. were randomly selected according to the centenarian number according to the protocol. A face-to-face interview was conducted to collect basic demographic characteristics, lifestyle, disease history and family history. Since vitamin D was first measured in 2011/2012 wave, we used this as the baseline survey and the most recent 2014 wave as the follow up survey.

### Study sample

**Fig. 1:**
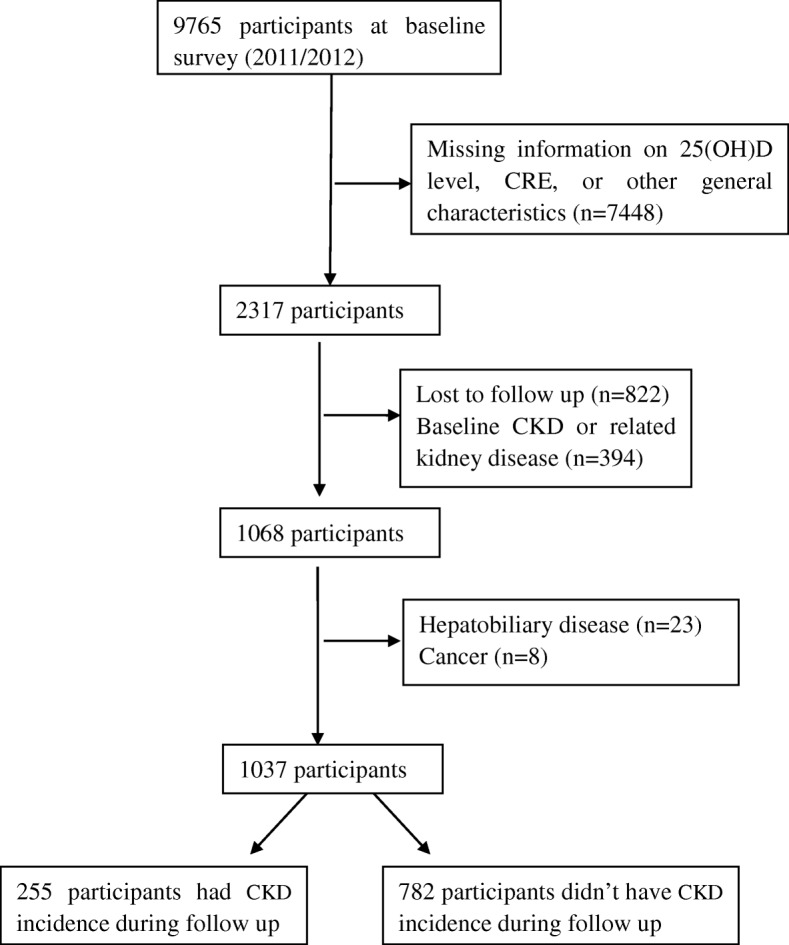
Flow chart of inclusion of participants

There were 9765 participants in 2011/2012 wave of the CLSHL. However, the blood biochemical tests were carried out only in 8 special areas-“The longevity village of China”. Excluding 7448 participants who didn’t have physical examination, there were 2317 participants. Those who had lost to follow up (*n* = 822), baseline CKD or related kidney disease (*n* = 394), hepatobiliary disease (*n* = 23), cancer (*n* = 8) were also excluded. The flow chart was presented in Fig. 1.

### Vitamin D

All blood samples were centrifuged within 1 h after collection, and the centrifuged specimens were placed in − 20° and transported to Beijing for uniform testing. Since 1,25(OH)_2_D were generated in renal proximal convoluted tubules, which were closely related with renal function, and 25(OH)D had relatively long half-life, so we used 25(OH)D as the suitable biomarker for assessing vitamin D deficiency or insufficiency, as most of epidemiological studies [4, 10]. The optimal vitamin D status was defined as serum 25(OH)D concentrations exceeding 75 nmol/L by the Endocrine Society [15]. According to the definition of endocrine society, serum 25 (OH) D levels were classified into three categories: sufficiency/optimal levels, more than 75 nmol/L; insufficiency, 50–75 nmol/L; deficiency, less than 50 nmol/ L. Also, for analysis, we divided 25(OH)D levels into quintiles: < 29.80 nmol/ L; 29.80–39.85 nmol/ L; 39.85–50.00 nmol/ L;50.0–62.90 nmol/ L; ≥62.90 nmol/ L.

### CKD

Albuminuria was defined as a urine albumin-creatinine ratio (ACR) > 30 mg/g. eGFR was calculated according to Chinese-modified CKD-EPI (cCKD-EPI) equation [16]. Impaired GFR was defined as eGFR< 60 ml/min/1.73m^2^. CKD was defined positive as either albuminuria or impaired GFR.

### Covariates

A standardized structured questionnaire was used to collect information, including demographic and sociological characteristics, lifestyle and disease history. Current married was classified into 5 categories (1 married and living with spouse; 2 married but not living with spouse; 3 divorced; 4 widowed; 5 never married;) and those answered 1 or 2 were classified into “yes”. Current smoking was classified into “yes” or “no” in the question “Do you smoke at the present time”. Current alcohol drinking was classified into “yes” or “no” in the question “Do you drink alcohol at the present time”. Current exercise was classified into “yes” or “no” in the question “Do you often participate in physical activities”. Previous diseases were self-reported including hypertension, diabetes, heart disease, and stroke/cerebrovascular disease.

### Statistical analysis

Data were expressed as mean ± standard deviation (SD) for continuous variable and n (%) for categorical variable. Baseline characteristics between those who had CKD incidence and those who didn’t develop CKD during follow up period were compared using independent t test for continuous variables and for categorical variables. Cox regression model was used to calculated the hazards ratio (HR) with 95%CI and assess the relationships between CKD (including both albuminuria and impaired GFR) and 25(OH)D level (in three types, including continuous, categorical, and quintiles). Related covariates were adjusted in the model, including age, gender, marital status, current smoking, current alcohol drinking, current exercise, baseline body mass index (BMI), albumin(ALB), urea nitrogen(BUN), creatinine(CRE), uric acid(SUA), baseline prevalence of hypertension, diabetes, heart disease and stroke. All analysis was conducted using SPSS 19.0(SPSS Inc., Chicago, IL).

## Results

The baseline characteristics of participants were shown in Table 1 and stratified by CKD new incident status. 1037 participants were included in the final analysis, and the mean age was 81.64 ± 12.33 years. The mean 25(OH)D was47.98 ± 22.00 nmol/L. The percentages of male, married, current smoking, current alcohol drinking and current exercise were 68.9, 50.6, 19.9 and 17.2% respectively. Compared with those who didn’t have CKD incidence, those who had new CKD incident had relatively longer older age, higher SBP, TC, LDL-C, BUN, CRE, SUA levels, and lower HDL-C, ALB, and 25(OH)D levels(*p* < 0.05).

**Table 1:** Baseline characteristics of participants according to CKD status at follow-up survey

Characteristics	CKD		*P*	Total (*n* = 1037)
	Yes (*n* = 255)	No (*n* = 782)		
mean ± SD				
Age (yrs)	85.44 ± 11.66	80.40 ± 12.30	< 0.001	81.64 ± 12.33
Height (cm)	154.81 ± 10.45	156.45 ± 10.72	0.031	156.04 ± 10.70
Weight (kg)	51.57 ± 12.17	53.86 ± 12.60	0.011	53.30 ± 12.53
BMI (kg/m^2^)	21.36 ± 3.90	21.84 ± 3.89	0.087	21.72 ± 3.89
SBP (mmHg)	146.50 ± 24.49	139.07 ± 21.51	< 0.001	139.28 ± 21.64
DBP (mmHg)	81.45 ± 12.53	81.27 ± 11.22	0.827	81.32 ± 11.55
TC (mmol/l)	4.21 ± 0.94	4.42 ± 0.95	0.002	4.37 ± 0.95
TG (mmol/l)	1.01 ± 0.59	1.02 ± 0.68	0.861	1.02 ± 0.66
HDL-C (mmol/l)	1.25 ± 0.36	1.32 ± 0.36	0.026	1.30 ± 0.36
LDL-C (mmol/l)	2.48 ± 0.77	2.65 ± 0.80	0.008	2.61 ± 0.79
FPG (mmol/l)	4.68 ± 1.63	4.59 ± 2.10	0.507	4.61 ± 1.99
ALB (g/L)	40.23 ± 5.04	41.13 ± 4.50	0.002	41.03 ± 4.65
BUN (mmol/L)	7.05 ± 2.00	6.55 ± 1.72	< 0.001	6.65 ± 1.80
CRE (μmol/L)	89.79 ± 28.29	74.69 ± 16.92	< 0.001	78.40 ± 21.53
SUA (μmol/L)	322.26 ± 96.22	278.14 ± 76.05	< 0.001	289.00 ± 8.36
25(OH)D (nmol/L)	37.63 ± 16.40	51.36 ± 22.54	< 0.001	47.98 ± 22.00
*n* (%)				
Male	124 (48.6)	383 (49.0)	0.923	507 (48.9)
Married	159 (62.4)	366 (46.8)	< 0.001	525 (50.6)
Current smoking	55 (21.6)	151 (19.3)	0.432	206 (19.9)
Illiteracy	95 (37.3)	366 (46.8)	0.008	461 (44.5)
Current alcohol drinking	47 (18.4)	131 (16.8)	0.537	178 (17.2)
Current exercise	31 (12.2)	127 (16.2)	0.115	158 (15.2)
Hypertension	90 (35.3)	182 (23.3)	< 0.001	272 (26.2)
Diabetes	22 (8.6)	52 (6.6)	0.287	74 (7.1)
Heart disease	21 (8.2)	64 (8.2)	0.979	85 (8.2)
Stroke	23 (9.0)	64 (8.2)	0.676	87 (8.4)

Data are mean ± SD for continuous values or % for category values

### Incidence of CKD according to baseline 25(OH)D level

There were a total of 255 CKD cases during the 21,586 person-years. The total three years’ incidence was 24.6% (95%CI: 22.0–27.2%). For those who were of vitamin D deficiency (less than 50 nmol/L) or insufficiency (50-75 nmol/L) at baseline, the CKD incidence was 32.7% (95%CI: 29.0–36.4%) and 14.2% (95%CI: 10.4–18.0%) respectively. For those who were of vitamin D sufficiency (more than 75 nmol/L), the CKD incidence was 7.8% (95%CI: 2.6–13.1%). Besides, as we can see from Table 2, the CKD incidence decreased along with quintiles of baseline 25(OH)D level; the first quintile had the highest incidence (44.4%) while the fifth had the lowest incidence (10.3%). For albuminuria and impaired eGFR, the incidence showed a similar trend (Table 2).

**Table 2:** Incidence of CKD according to baseline 25(OH)D level

Variable	Classifications of baseline 25(OH)D level			Quintiles of baseline 25(OH)D level					Total
	< 50 nmol/L	50–75 nmol/L	≥75 nmol/L	≤30 nmol/L	30–40 nmol/L	40–50 nmol/L	50-63 nmol/L	>63 nmol/L	
Albuminuria									
Number of incident cases	109	10	3	53	35	21	7	6	122
Incidence (%)	17.6 (14.6–20.6)	3.2 (1.2–5.1)	2.9 (0.3–5.6)	25.6 (19.7–31.6)	16.2 (11.3–21.1)	10.8 (6.4–15.1)	3.6 (1.0–6.2)	2.7 (0.6–4.8)	11.8 (9.8–13.7)
Total person-years	1895	1012	328	632	661	605	622	715	3253
Incidence rate (per 100 person years)	5.8 (4.8–6.9)	1.0 (0.6–1.8)	0.9 (0.3–2.8)	8.4 (6.4–11.0)	5.3 (3.8–7.4)	3.5 (2.3–5.3)	1.1 (0.5–2.4)	0.8 (0.4–1.8)	3.8 (3.1–4.5)
Impaired eGFR									
Number of incident cases	117	36	6	48	41	28	24	18	159
Incidence (%)	18.9 (15.8–22.0)	11.4 (7.9–14.8)	5.9 (1.3–10.4)	23.2 (17.4–28.9)	19.0 (13.8–24.2)	14.4 (9.4–19.3)	12.2 (7.9–15.3)	8.1 (4.5–11.6)	15.3 (13.1–17.5)
Total person-years	1884	975	322	639	651	593	600	698	3181
Incidence rate (per 100 person years)	6.2 (5.2–7.4)	3.7 (2.7–5.2)	1.9 (0.8–4.1)	7.5 (5.7–9.7)	6.3 (4.6–8.6)	47 (3.3–6.8)	4.0 (2.7–6.0)	2.6 (1.6–4.1)	5.0 (4.3–5.8)
CKD									
Number of incident cases	202	45	8	92	66	44	30	23	255
Incidence (%)	32.7 (29.0–36.4)	14.2 (10.4–18.0)	7.8 (2.6–13.1)	44.4 (37.7–51.2)	30.6 (24.4–36.7)	22.6 (16.7–28.4)	15.3 (10.3–20.3)	10.3 (6.3–14.3)	24.6 (22.0–27.2)
Total person-years	1850	967	318	621	645	585	596	689	3135
Incidence rate (per 100 person years)	10.9 (9.5–12.5)	4.7 (3.5–6.2)	2.5 (1.3–5.0)	14.8 (12.1–18.2)	10.2 (8.0–13.0)	7.5 (5.6–10.1)	5.0 (3.5–7.2)	3.3 (2.0–5.0)	8.1 (7.2–9.2)

### HRs and 95% CI of albuminuria, eGFR decrease and CKD incidence according to baseline 25(OH)D levels

Table 3 showed the HRs of baseline 25(OH)D levels for albuminuria, impaired eGFR and CKD incidence. In the Cox model, after adjusted for age, gender, marital status, current smoking, current alcohol drinking, current exercise, baseline BMI, ALB, BUN, CRE, SUA, baseline prevalence of hypertension, diabetes, heart disease and stroke in the model, the HRs of baseline 25(OH)D levels for albuminuria, impaired eGFR and CKD incidence were 0.952(95%CI: 0.941–0.963), 0.975(95%CI: 0.966–0.983), and 0.966(95%CI: 0.959–0.973) respectively. When use the classifications (sufficiency, insufficiency, deficiency) or quintiles of baseline 25(OH)D levels in the Cox model, the corresponding HRs showed an increasing trend along with the decrease of baseline 25(OH)D levels (p for trend < 0.001) (Table 3) .

**Table 3:** HRs and 95% CI of albuminuria, eGFR decrease and DKD incidence according to baseline 25(OH)D level

	Variable type	HR* (95%CI)	*P*
Albuminuria	Continuous variable	0.952 (0.941–0.963)	< 0.001
	Classifications		< 0.001
	< 50 nmol/L	5.737 (2.807–11.725)	
	50–75 nmol/L	1.695 (0.684–4.200)	
	≥ 75 nmol/L	1.00 (Ref)	
	Quintiles		< 0.001
	≤ 30 nmol/L	7.864 (4.012–14.717)	
	30–40 nmol/L	5.631 (2.824–11.228)	
	40–50 nmol/L	3.328 (1.571–7.050)	
	50-63 nmol/L	1.302 (0.485–3.495)	
	>63 nmol/L	1.00 (Ref)	
Impaired eGFR	Continuous variable	0.975 (0.966–0.983)	< 0.001
	Classifications		< 0.001
	< 50 nmol/L	4.329 (2.015–9.300)	
	50–75 nmol/L	2.068 (0.920–4.649)	
	≥ 75 nmol/L	1.00 (Ref)	
	Quintiles		< 0.001
	≤ 30 nmol/L	4.645 (2.808–7.684)	
	30–40 nmol/L	4.120 (2.460–6.900)	
	40–50 nmol/L	2.750 (1.598–4.732)	
	50-63 nmol/L	2.010 (1.156–3.495)	
	>63 nmol/L	1.00 (Ref)	
CKD	Continuous variable	0.966 (0.959–0.973)	0.003
	Classifications		< 0.001
	< 50 nmol/L	4.667 (2.285–9.532)	
	50–75 nmol/L	1.811 (0.909–3.608)	
	≥ 75 nmol/L	1.00 (Ref)	
	Quintiles		< 0.001
	≤30 nmol/L	5.677 (3.771–8.546)	
	30–40 nmol/L	4.410 (2.892–6.724)	
	40–50 nmol/L	2.832 (1.810–4.431)	
	50-63 nmol/L	1.821 (1.138–2.914)	
	>63 nmol/L	1.00 (Ref)	

Adjusted for age, gender, marital status, illiteracy, current smoking, current alcohol drinking, current exercise, baseline BMI, ALB, BUN, CRE, SUA, baseline prevalence of hypertension, diabetes, heart disease and stroke

## Discussion

In this population based cohort study with a large sample, 25(OH)D deficiency was independently associated with CKD (including both albuminuria and impaired eGFR). The correlation between baseline 25 (OH) D levels and CKD incidence was most pronounced among the lowest quintile. The trend for the observed linear relationship between baseline 25(OH)D levels and CKD incidence persisted with additional adjustment for related covariates.

The association between 25(OH)D deficiency and albuminuria incidence was demonstrated in previous studies. The results based on 10,732 adults from the AusDiab (Australian Diabetes, Obesity and Lifestyle) study showed that vitamin D deficiency (25(OHD) level < 50nnol/L) was significantly associated with albuminuria prevalence in the multivariate mode, and the OR was 1.54(95%CI: 1.14–2.07) [4]. The results based on 15,068 adults from NHANES III (the Third National Health and Nutrition Examination Survey) showed that low 25(OH)D levels were associated with an increased prevalence of albuminuria in U.S. population [10]. The association was also demonstrated in a multicenter CKD cohort-Study of Early Evaluation of Kidney Disease, where low 25(OH)D levels were independently associated with albuminuria [17]. Our observations were in keeping with these findings, which added prospective evidence about the association between vitamin D and proteinuria.

Relationships between 25 (OH) D levels and impaired GFR were inconclusive. There showed no significant associations in multivariate models in neither AusDiab study nor NHANESIII data [4, 10]. Contrary to the results of cross-sectional study, the results of the cohort study demonstrated an association between 25 (OH) D levels and GFR progression. In a small sample cohort consisted of stages 2–5 CKD patients, serum 25 (OH) D predicted both time to death and end stage renal disease [18]. Another community-based cohort study also showed that lower 25(OH)D was associated with increased risk of GFR loss [12]. There were many reasons about this inconsistency for associations between cross-sectional studies and cohort studies. One may be attributable to the fact that vitamin D levels varies by people and were associated with season and sunlight [12]. Also,there existed another possible mechanism, some of CKD patients do not progress, [19]which indicated that 25 (OH) D deficiency may be an indicator for distinguish from those who were at higher risk of progression and those who were not. However, there was no evidence about whether there was an association between 25(OH)D and impaired GFR incidence among normal participants who were free of CKD at baseline, and our results showed that lower 25(OH)D was associated with impaired GFR incidence based on cohort study. The results added evidence that 25 (OH) D deficiency might be used as an indicator to distinguish from those who were at higher risk of impaired GFR incidence and those who were not.

The protective effect of vitamin D on CKD may be attributed to a set of mechanisms [20–22]. First, there was a series of evidence showed that the protective effect of vitamin D on renin can be treated by RAS and NK-kB. Vitamin D analogues would reduce the expression of renin and thus decrease the expression of angiotensin II, which is the key mediator of proteinuria and renal damage. Besides, low vitamin D levels were linked to higher expression of inflammatory parameters. Also, vitamin D levels might be associated with hypertension, impaired glucose metabolism, which was also correlated with CKD.

There were several strengths of this study. All the participants were recruited from a national level, and all interview and examination process was standardized. Second, different from previous studies, we observed not only albuminuria, but also impaired eGFR incidence among those who were free of CKD at baseline. Also, our study has several limitations. First, baseline 25 (OH) D was measured only once, which maybe unstable and fluctuated by related factors. Second, since biomedical tests were only done on a subset of the sample (8 special areas-“The longevity village of China”), there may be some selection bias-imbalance between those included and those not (Additional file 1: Table S1). We compared the baseline characteristics between the two groups, those include had higher BMI, SBP, and 25 (OH) D levels. Third, considering all participants were elderly, the results may not be applicable to adults. Fourth, due to the limitation of field survey, the CLHLS study didn’t have information on the treatment with vitamin D (drug treatment or intake of nutrients containing vitamin D), vitamin D related indicators tested by blood biochemical tests (parathyroid hormone or phosphate), other confounding unmeasured factors which might have impact on serum 25(OH)D levels. Further cohort study with wide age and longer follow up years were needed.

## Conclusions

In summary, this study demonstrated there was a strong, independent, linear association between baseline lower 25(OH)D level and CKD (including both albuminuria and impaired GFR) incidence. Given the evidence from basic research, it is tempting to speculate that vitamin D deficiency may present a novel indicator for CKD incidence and progression.

## Additional file

Additional file 1: **Table S1.** Baseline characteristics of participants included in the analysis and those excluded. (DOC 45 kb)

## Abbreviations

ALT: Alanine aminotransferase BMI body mass index CI Confidence interval CKD Chronic kidney disease CLHLS Chinese Longitudinal Healthy Longevity Survey DBP Diastolic blood pressure DR Diabetic retinopathy FBG Fasting plasma glucose eGFR Estimated glomerular filtration HDL-C High density lipoprotein cholesterol HR Hazard ratio LDL-C Low density lipoprotein cholesterol SBP Systolic blood pressure TBiL Bilirubin TC Total cholesterol TG Triglyceride 2hPG Two hours plasma glucose;
