# Supplementary material for: Serum 25-Hydroxyvitamin D were associated with higher risk of both albuminuria and impaired GFR incidence: a cohort study based on CLHLS study
Source: BMC Nephrol. 2019 Jan 15;20:20. doi: 10.1186/s12882-019-1202-8 (PMC6332878; doi:10.1186/s12882-019-1202-8)
Supplement: Supplementary file 1 — Table S1. Baseline characteristics of participants included in the analysis and those excluded. (DOC 45 kb) [file 12882_2019_1202_MOESM1_ESM.doc]

Additional file 1: Table S1 Baseline characteristics of participants included in the analysis and those excluded

| Characteristics | Included  (n=1037) | Excluded (n=8728) | P | Total  (n=9765) |
| --- | --- | --- | --- | --- |
| mean±SD |  |  |  |  |
| Age (yrs) | 81.64±12.33 | 86.32±11.15 | <0.001 | 85.78±11.41 |
| Height(cm) | 156.04±10.70 | 155.02±10.83 | 0.179 | 155.08±10.83 |
| Weight(kg) | 53.30±12.53 | 51.00±12.15 | <0.001 | 51.23±12.26 |
| BMI(kg/m^2^) | 21.72±3.89 | 21.23±4.17 | 0.001 | 21.30±4.14 |
| SBP(mmHg) | 139.28±21.64 | 136.23±21.52 | <0.001 | 136.91±21.73 |
| DBP(mmHg) | 81.32±11.55 | 80.33±13.70 | 0.003 | 80.50±13.45 |
| % |  |  |  |  |
| Male | 507(48.9) | 3891(44.6) | 0.021 | 4398(45.0) |
| Married | 525(50.6) | 5542(63.5) | <0.001 | 6067(62.1) |
| Current smoking | 206(19.9) | 1227(14.1) | 0.439 | 1733(17.7) |
| Current alcohol drinking | 178(17.2) | 1462(16.8) | 0.058 | 1640(16.8) |
| Current exercise | 158(15.2) | 3023(34.6) | <0.001 | 3181(32.6) |
| Hypertension | 272(26.2) | 2480(28.4) | 0.505 | 2752(28.2) |
| Diabetes | 74(7.1) | 397(4.5) | <0.001 | 471(4.8) |
| Heart disease | 85(8.2) | 1090(12.5) | 0.875 | 1175(12.0) |
| Stroke | 87(8.4) | 715(8.2) | 0.589 | 802(8.2) |

Data are mean±SD for continuous values or % for category values;
